# Slab Waveguide and Optical Fibers for Novel Plasmonic Sensor Configurations

**DOI:** 10.3390/s17071488

**Published:** 2017-06-24

**Authors:** Nunzio Cennamo, Francesco Mattiello, Luigi Zeni

**Affiliations:** Department of Industrial and Information Engineering, University of Campania “*L. Vanvitelli*”, via Roma 29, 81031 Aversa, Italy; francesco.mattiello@unicampania.it (F.M.); luigi.zeni@unicampania.it (L.Z.)

**Keywords:** optical sensors, PMMA waveguides, plastic optical fibers, surface plasmon resonances, holder for plasmonic chips, refractive index sensing, slab waveguides

## Abstract

The use of plasmonic sensor devices often requires replaceable parts and disposable chips for easy, fast and on-site detection analysis. In light of these requests, we propose a novel low-cost surface plasmon resonance sensor platform for possible selective detection of analytes in aqueous solutions. It is based on a Polymethyl methacrylate (PMMA) slab waveguide with a thin gold film on the top surface inserted in a special holder, designed to produce the plasmonic resonance at the gold-dielectric interface. A wide-band light is launched in the PMMA slab waveguide through a trench realized in the holder directly, and illuminated with a PMMA plastic optical fiber (POF) to excite surface Plasmon waves. The output light is then collected by another PMMA POF kept at the end of the slab at an angle of 90° to the trench, and carried to a spectrometer. In this configuration, the trench has been used because a large incident angle is required for surface plasmon resonance excitation. The preliminary results showed that the sensor’s performances make it suitable for bio-chemical applications. The easy replacement of the chip allows for the production of an engineered platform by simplifying the measurement procedures.

## 1. Introduction

A very sensitive method for determining refractive index variations at the interface between a metal layer or nanoparticles and a dielectric medium is based on Surface Plasmon Resonance (SPR) or Localized Surface Plasmon Resonance (LSPR). In the scientific literature, several review papers describe SPR and LSPR sensors and their applications [1,2,3]. As an example, plasmonic works based on nanoparticles or nanocomposite thin silver films can be found in references [4,5,6,7].

In many works, the SPR sensors use a highly refractive index prism coated with a thin metallic layer (or a glass chip coated with a thin layer of metal and bonded to the prism), a polarizer and an array of detectors. For example, Texas Instruments Inc. has developed Spreeta, a low-cost and portable electronic system based on this approach [8]. 

Generally, in prism-based Kretschmann configurations, to satisfy the plasmonic condition, the angle of the incident light changes when the surrounding medium refractive index changes. Nevertheless, as the sensors are usually bulky and require expensive optical equipment, it is not easy to miniaturize them and, in addition, the remote sensing capability may be difficult to implement. 

Jorgenson et al. have realized the SPR sensor in optical fiber, without prisms [9]. Usually, the metal layer is deposited on the core of the optical fiber directly. The SPR sensors in optical fiber allow for a remote sensing and may reduce the dimensions and price of the sensor system. At the beginning, the optical fibers employed were made of glass, but recently, plastic or specialty optical fibers have been used [10,11,12,13,14,15,16,17,18,19,20,21]. The plastic optical fibers (POFs) present exceptional flexibility, simple manipulation, great numerical aperture, large diameter, and are able to withstand smaller bend radii than glass fibers.

Therefore, the POFs are particularly advantageous for realizing low-cost SPR sensors. Usually, the SPR optical fiber sensors show a noticeably high sensitivity [14,15,16,17,18,19,20], due to the fact that they are able to detect even small variations of refractive index of the medium (dielectric) in contact with the metal layer, where biological or artificial receptors are present. They selectively recognize the analyte present in the liquid under test, producing a local variation of the dielectric’s refractive index in contact with the metal film. 

In this work, we have developed a novel configuration for a low-cost SPR sensor. This configuration is based on two POFs, a removable PMMA chip with gold film on the top and a new special holder designed to excite the plasmonic resonance at the gold-dielectric interface. 

The preliminary experimental results have shown that the performances of this sensor are comparable to those obtained with the SPR-POF sensor described in [21], with some benefits. In particular, this novel approach doesn’t require the polishing procedures of the POFs and, moreover, the slab waveguide chip is very easy to use in order to potentially realize, on its surface, plasmonic nanostructures by lithography process and for production, on the industrial scale, of disposable SPR chips with different receptors. 

The special holder is designed to fix all the optical components, and to ensure the reproducibility of the results, when the removable components are introduced into, and removed from, the system. Five replicated measurements of the sensor response were performed to assess the repeatability of the results.

## 2. Materials and Methods

### 2.1. Theoretical Model

In this section, we recall the approximate theoretical model, already described and used for multimode waveguides [18,19,20,21,22,23]. All the parameters of the waveguide influence the transmittance form of the outgoing light. In this model, we assume two hypotheses:

(a) Along the waveguide, the angular power distribution of the source (*P*_0_) is uniform;
(b) The distribution of the TE and TM polarization state is equal.

In these hypotheses, the power at the end of waveguide can be calculated as:

(1) $$P_{out}(\lambda ,n_{sen\sin g})=\frac{1}{2}(\int_{\theta_{critical}}^{90{}^{\circ}}R_{p}^{N_{R}}P_{0}(\lambda ,\theta ,n_{sen\sin g})d\theta +\int_{\theta_{critical}}^{90{}^{\circ}}R_{s}^{N_{R}}P_{0}(\lambda ,\theta ,n_{sen\sin g})d\theta )$$

where *P*_0_ is the power of the source, *R_p_* and *R_s_* are the reflectance coefficients and *N_R_* indicates the number of reflections in the sensing region (*N_R_* is a function of the angle *θ* and of the sensing region size: length (L) and width (W)). As shown in Equation (1), the spectral power at the end of the optical waveguide is the collected physical quantity.

In Equation (1), *R_p_* and *R_s_* can be calculated by the transfer matrix formalism [18,19,20,21,22,23]. In particular, they can be calculated by the *N*–layer model [18] as:

(2) $$R_{p/s}={\left|\frac{\left(M_{11}^{p/s}+M_{12}^{p/s}\xi_{N}^{p/s}\right)\xi_{0}^{p/s}-\left(M_{21}^{p/s}+M_{22}^{p/s}\xi_{N}^{p/s}\right)\xi_{N}^{p/s}}{\left(M_{11}^{p/s}+M_{12}^{p/s}\xi_{N}^{p/s}\right)\xi_{0}^{p/s}+\left(M_{21}^{p/s}+M_{22}^{p/s}\xi_{N}^{p/s}\right)\xi_{N}^{p/s}}\right|}^{2}$$

In Equation (2), $\xi_{k}^{p/s}$ is the optical admittance, whereas *M_nm_* are the elements of the characteristic matrix of the layered system defined as follows [18]:

(3) $$\left[M\right]=\left[\begin{array}{cc} M_{11} & M_{12}\\ M_{21} & M_{22} \end{array}\right]=\prod_{K=1}^{N-1}\left(\left[\begin{array}{cc} \cos\varsigma_{k} & \frac{-i\sin\varsigma_{k}}{\xi_{k}}\\ -i\xi_{k}\sin\varsigma_{k} & \cos\varsigma_{k} \end{array}\right]\right)$$

This model well describes the real situation, but it doesn’t consider mode coupling phenomena and the scattering, diffraction or dispersion caused by the roughness of the layer. We have recalled, in Equations (1)–(3), the main parameters of the approximate numerical model (fully described in [18]) used to design the geometrical parameters of the slab waveguide based on a PMMA layer and a gold film. 

In this work, we have used normalized transmitted power (*T*(*λ*)), defined as the ratio of the power at the end of waveguide and the reference power:

(4) $$T(\lambda )=\frac{P_{out}(\lambda )}{P_{ref}(\lambda )}$$

where the reference power *P_ref_*(*λ*) is obtained when the air is in contact with the gold film. In fact, when air is on the gold surface, the SPR phenomenon is not present in this platform and, consequently, this spectrum can be used as the reference. 

For the comparative analysis between this new sensor and similar SPR sensors, we have used three parameters: sensitivity (*S*), resolution (Δ*n*) and SNR (signal to noise ratio). 

Finally, we want to recall how these parameters are defined in the spectral mode configuration (white light source/Spectrometer) [18,19,20,21,22,23]. 

The SPR sensor’s sensitivity (*S*) can be defined as [18,19,20,21,22,23]:

(5) $$S(n_{sen\sin g})=\frac{\delta \lambda_{resonance}}{\delta n_{sen\sin g}}$$

In other words, the sensitivity is the variation of the *T(λ)* curve dip (resonance wavelength) related to the unit change in refractive index. In fact, when the refractive index value of the sensing layer (*n_sensing_*) changes the dip of *T*(*λ*), namely resonance wavelength (*λ_resonance_*), shifts.

The resolution (Δ*n*) of the sensor is the smallest measurable change in refractive index. In bio and chemical sensing, the minimum resolution required on the metal surface is around 10^−5^ RIU [13,24]. The SPR sensitivity decreases exponentially when the distance from the gold surface increases. In these preliminary results, without a receptor on the gold surface, we have tested, with different water-glycerine solutions, the “bulk sensitivity” of the novel SPR sensor in order to compare this new platform with the previously developed SPR sensor platform based on a *D*-shaped POF [21]. 

In spectral configuration, the resolution (Δ*n*) depends on the spectrometer’s spectral resolution (*δλ_DR_*) [18,19,20,21,22,23], as defined in the equation:

(6) $$\Delta n(n_{sen\sin g})=\frac{\delta n_{sen\sin g}}{\delta \lambda_{resonance}}\delta \lambda_{DR}=\frac{1}{S(n_{sen\sin g})}\delta \lambda_{DR}$$

In the end, the signal to noise ratio (SNR) of the sensor can be defined as [18,19,20,21,22,23]:

(7) $$SNR\left(n_{sensing}\right)=\frac{\delta \lambda_{resonance}}{\delta FWHM}$$

where *δ_FWHM_* is the variation of the full width at half maximum (*FWHM*) of the *T*(*λ*) curve. 

### 2.2. SPR Sensor Configuration

**Figure 1:**
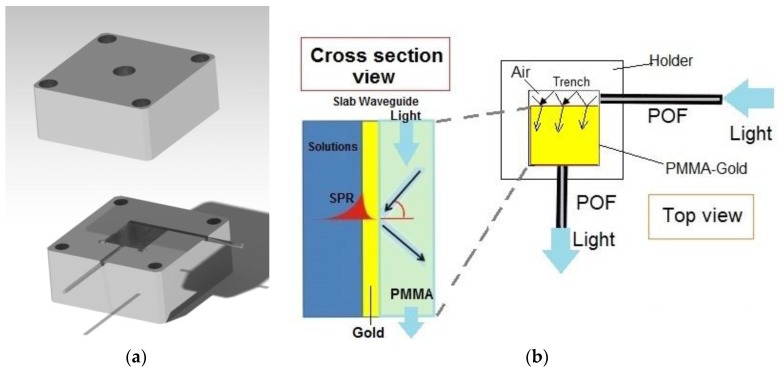
SPR sensor system: (**a**) Aluminum holder developed for removable SPR-chip and POFs (**b**) Schematic view of the light path in the SPR sensor (Top and Cross section view of the sensor system outline).

The SPR sensor system has been obtained by a removable PMMA layer with a gold film on the top, two POFs and a special holder designed to generate the SPR in the PMMA-gold multilayer. Figure 1a shows the developed sensor system. In particular, the removable chip is a PMMA layer, 0.5 mm thick and 10 mm × 10 mm in size, with a thin gold film on the top (60 nm thick). The POFs have a core of PMMA (980 μm) and a cladding of fluorinated polymer (20 μm). The refractive index, in the visible range of interest, is about 1.49 for PMMA, 1.41 for the fluorinated polymer. The special holder has been obtained with two aluminum blocks, the first one to insert the PMMA chip and the two POFs (see Figure 1a) and the second one as a cover, equipped with a hole and an o-ring to retain the liquid samples. For bio and chemical sensing applications, we could substitute this block for a flow cell (thermo-stabilized).

Figure 1b shows a schematic view of the light path in the SPR sensor. As indicated in Figure 1b, the exciting light is introduced in the PMMA waveguide by a 10 mm long trench (size: 1 mm × 1 mm), realized in the holder, illuminated by a POF (1 mm of total diameter). On the other hand, another PMMA POF kept at the end of the PMMA waveguide at a 90° angle to the trench, is exploited to carry the output light to a spectrometer. The trench “air waveguide” has been designed because a large incident angle (*θ*) is required for surface plasmon resonance excitation.

**Figure 2:**
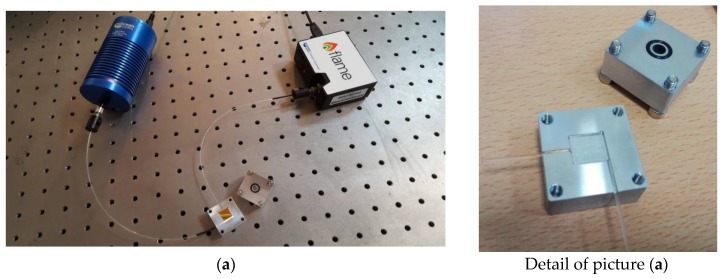

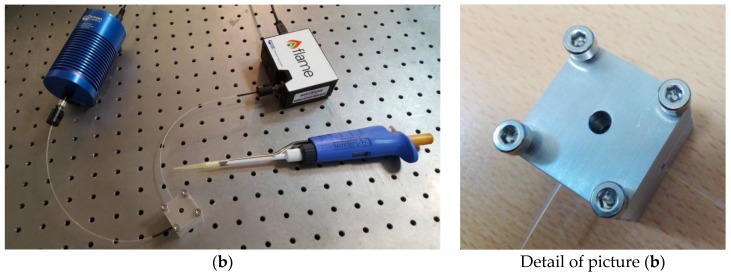
Experimental setup: (**a**) Setup with the opened aluminum holder and the removable SPR-chip: the trench and the o-ring are clearly visible; (**b**) Setup with the aluminum holder sealed to allow measuring the refractive index of a liquid sample by dropping it through the top hole.

Figure 2 shows the experimental setup. It is composed by a halogen lamp (HL-2000-LL, Ocean Optics, Dunedin, FL, USA) exhibiting a wavelength emission range from 360 nm to 1700 nm, as the light source, the POFs-PMMA chip waveguide and a spectrometer (FLAME-S-VIS-NIR-ES, Ocean Optics, Dunedin, FL, USA) connected to a PC. The spectrum analyzer detection range is from 350 nm to 1023 nm and the spectral resolution (*δλ_DR_*) of the spectrometer is 1.5 nm (FWHM).

## 3. Experimental Results

**Figure 3:**
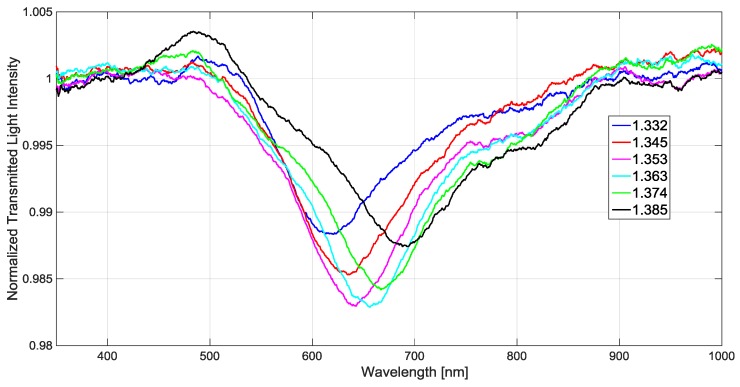
For six different refractive indices of the solutions, experimental SPR spectra (normalized to the reference spectrum).

In the experimental measurements, to obtain solutions with different refractive indices, we used six water-glycerin solutions. An Abbe refractometer was used to prepare and characterize these solutions. In order to maintain the solutions at a constant refractive index, we stored them at 20 °C. Figure 3 shows the SPR spectra (*T*(*λ*)) for the water-glycerin solutions mentioned above (refractive index from 1.332 to 1.385). SPR transmission spectra were normalized to the reference spectrum, achieved with air as the surrounding medium [20,21].

**Figure 4:**
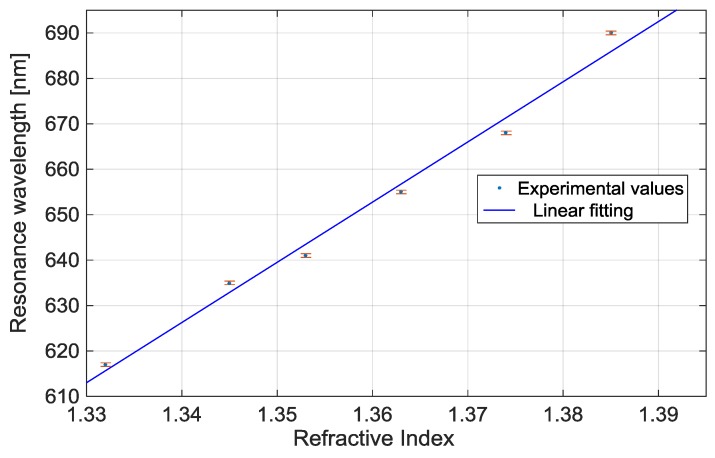
Resonance wavelength versus refractive index of the aqueous medium and linear fitting of the experimental values.

Figure 4 shows the resonance wavelength (reported in Figure 3) versus the refractive index together the linear fitting to the experimental values. This linear fitting shows a good linearity with a 0.99 Pearson’s correlation coefficient (R). Each experimental value is the average of 3 subsequent measurements and the respective standard deviations (error bars) are also shown.

The sensitivity, as indicated in Equation (5), is the shift of the resonance wavelength per unit change in refractive index. Hence, it can be derived from the slope of the linear fitting shown in Figure 4. In this case it is about 1.3 × 10^3^ (nm/RIU).

The sensor’s resolution (Δ*n*) can be calculated, as indicated in Equation (6), by the reciprocal of sensitivity multiplied to the resolution of the spectrometer (δ*λ*_DR_):

(8) $$\Delta n=\frac{1}{S}\cdot \delta \lambda_{\mathrm{DR}}=\frac{1.5\;\left[\mathrm{nm}\right]}{1.3\times 10^{3}\left[\frac{\mathrm{nm}}{\mathrm{RIU}}\right]}=1.1\times 10^{-3}\;\left[\mathrm{RIU}\right]$$

The SPR resolution increases exponentially when the distance from the sensing surface increases. The calculated average value of the resolution (see Equation (8)) is appealing for a large number of bio and chemical applications. In fact, in these cases a “bulk resolution” value (∆*n*) of about 10^−3^ is required, as demonstrated by SPR-POF sensor platform [21,25] already used in several biochemical applications [26,27,28]. We have designed this new sensor system platform to obtain this specific characteristic.

Finally, it is important to underline that, when a bio-receptor is present on the gold film, a specific protocol to measure the changes in a refractive index resulting from bio-interaction, under the same conditions, is implemented [29]. In particular, a washing step with a “buffer” solution was carried out after the receptor-analyte binding and the spectrum was recorded only afterward. This means that a washing step is necessary after each addition of the sample being tested in order to measure the real shift of the resonance determined by the receptor-analyte interaction on the sensing surface.

## 4. Discussion

We have presented a novel SPR sensor configuration based on a special holder, two POFs and a PMMA’s chip. The experimental results showed that the sensor’s performance is similar to that obtained with D-shaped POF-SPR sensors (1000 μm diameter POF) [21]. Table 1 shows the average values of the performance parameters, obtained with external medium refractive index ranging from 1.332 to 1.385, for the D-shaped POF-SPR sensor [21] and the new SPR sensor system.

Finally, we have analyzed the SNR parameter (Equation (7)), which is dependent on the refractive index. For this configuration, the full width at half maximum (FWHM) of the SPR curve (see Figure 3), for external refractive indices from 1.332 to 1.385, are 120 ÷ 150 nm. These experimental values are smaller than the corresponding parameters in the POF-SPR sensor [21,25], thus leading to a better SNR.

**Figure 5:**
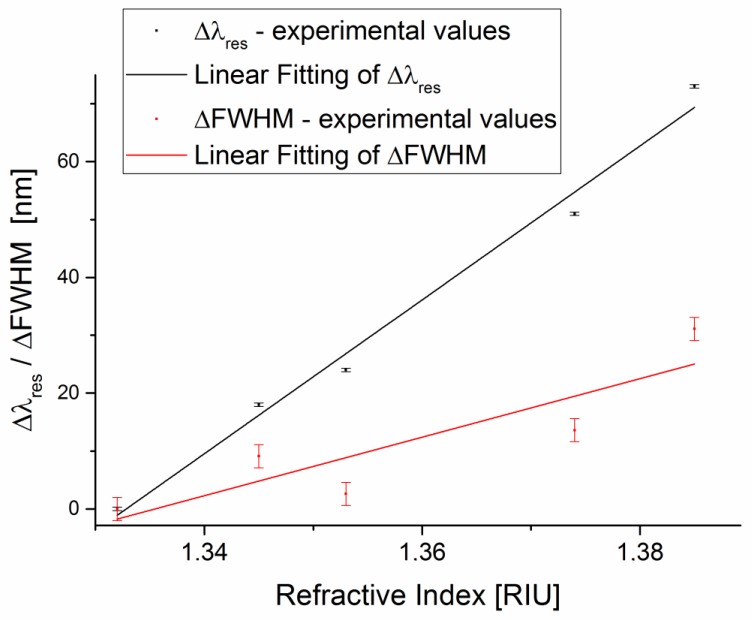
Experimental resonance wavelength shift and FWHM shift, with respect to water solution (1.332 RIU), versus refractive index. The error bars represent the standard deviation of the experimental values.

Figure 5 shows the resonance wavelength shift and FWHM shift versus the refractive index, and the error bars represent the standard deviation. The resonance wavelength shift (∆*λ_res_*) and FWHM shift (∆FWHM) have been calculated with respect to water solution (1.332 RIU) by Matlab software. In the same figure, we report the linear fitting to the experimental data. The parameters of both linear fittings are reported in Table 2.

Therefore, in this range, the average value of the SNR is 2.63. This value can be determined by employing a first-order approach, according to Equation (7), as the ratio of ∆*λ_res_* and ∆FWHM slope (shown in Figure 5).

The experimental results demonstrate that this new sensor configuration exhibits good performance for the SNR, too [21,25]. The linear fitting used in this approach does not imply an actual linear relationship but it is just a way to extrapolate a trend and allow an easy comparison between the different SPR sensor systems.

## 5. Conclusions

A novel low-cost, easy-to-realize and small-sized sensor system, based on SPR excited by PMMA chip and POFs, has been realized and experimentally tested. The proposed sensor system is based on a special holder designed to produce the excitation of surface plasmons at the interface between the medium being tested and a thin gold layer deposited on a PMMA slab waveguide. The interesting aspect is that the cost of the removable SPR sensor chip is in the order of a few Euro, and the cost of the interrogation unit is less than 3000 euro. This cost could even decrease, if production on industrial scale were considered.

The experimental results have demonstrated good performances in terms of sensitivity, resolution and SNR. In particular, these results are similar to those obtained by the classic configuration reported in the literature, but the advantages of this approach are the possibility of remote sensing, by POFs, with a removable chip sensor for the production of an engineered platform and a new holder useful for thermo-stabilized flow cell in biochemical sensing applications. 

## Figures and Tables

**Table 1:** Performance comparison between POF-SPR sensor [21] and PMMA SPR chip sensor.

Sensor	(S) Sensitivity (nm/RIU)	(∆*n*) Resolution (RIU)
PMMA SPR chip sensor	1.330 × 10^3^	1.1 × 10^−3^
POF-SPR sensor [18]	1.325 × 10^3^	1.1 × 10^−3^

**Table 2:** Linear fitting parameters of the experimental values ∆*λ_re_*_s_ and ∆FWHM (shown in Figure 5).

Experimental Data	Slope (nm/RIU)	R^2^
∆*λ_res_*	1330	0.98
∆_FWHM_	505	0.71
